# Formation of Racemic Phases of Amino Acids by Liquid-Assisted Resonant Acoustic Mixing Monitored by Solid-State NMR Spectroscopy

**DOI:** 10.3390/molecules30183745

**Published:** 2025-09-15

**Authors:** Leeroy Hendrickx, Calogero Quaranta, Emilian Fuchs, Maksim Plekhanov, Mirijam Zobel, Carsten Bolm, Thomas Wiegand

**Affiliations:** 1 Max Planck Institute for Chemical Energy Conversion, Stiftstr. 34–36, 45470 Mülheim an der Ruhr, Germany; hendrickx@itmc.rwth-aachen.de; 2 Institute of Technical and Macromolecular Chemistry, RWTH Aachen University, Worringerweg 2, 52074 Aachen, Germany; emilian.fuchs@rwth-aachen.de; 3 Institute of Organic Chemistry, RWTH Aachen University, Landoltweg 1, 52074 Aachen, Germany; calogero.quaranta@rwth-aachen.de; 4 Institute of Crystallography, RWTH Aachen University, Jägerstraße 17–19, 52066 Aachen, Germany; plekhanov@ifk.rwth-aachen.de (M.P.); zobel@ifk.rwth-aachen.de (M.Z.)

**Keywords:** amino acids, mechanochemistry, resonant acoustic mixer, solid-state nuclear magnetic resonance, solvents

## Abstract

Mechanochemistry has become a fundamental method in various sciences including biology and chemistry. Despite its popularity, the mechanisms behind mechanochemically induced reactions are not very well understood. In previous work, we investigated molecular-recognition processes of molecules capable of forming racemic phases in ball mill devices. Solid-state nuclear magnetic resonance (solid-state NMR) was used as the key technique to analyze such events. We now extended this study and focused on mechanochemically induced racemic-phase formations of two representative amino acids, alanine and serine, in a resonant acoustic mixer. The data reveal the importance of adding small amounts of solvents (here water) to facilitate the underlying solid-state molecular-recognition processes. The role of water therein is further studied by deuterium magic-angle spinning (MAS) NMR experiments, also revealing that resonant acoustic mixing (RAM) enables efficient hydrogen to deuterium exchange in enantiopure serine, paving the way to deuterate organic compounds in the RAM device.

## 1. Introduction

Solid-state NMR spectroscopy is an important method for studying structure and dynamics in a variety of materials, comprising catalysts, pharmaceutics, battery materials, polymers, and biomaterials to mention only a few (for some selected review articles see [1,2,3,4,5]). Particularly, it offers various benefits in mechanochemistry, since the obtained reaction products can directly be analyzed and characterized without the need of using solvents for work-up or purification, which could eventually alter the product composition [6,7,8]. Evaluating molecular-recognition events by solid-state NMR spectroscopy is promising, because the NMR chemical-shift values, for instance, are highly sensitive to noncovalent interactions essential in such processes, including hydrogen bonds or dispersion interactions [9]. Furthermore, solid-state NMR is capable of distinguishing enantiopure and racemic crystalline phases, which is impossible to achieve in solution [10]. To gain such knowledge on the solid-state behavior has relevance in biological settings for resolutions for chiral drugs, for example [11].

In recent years, mechanochemistry has gained significant attention in a variety of fields [12,13,14,15,16,17]. Although, in organic chemistry, many useful mechanochemical transformations have been discovered, a fundamental understanding of the underlying processes is still missing. This is unfortunate, because an improved mechanistic knowledge could eventually lead to a more targeted search for future applications. To fill this gap, we utilized the unique opportunities offered by solid-state NMR spectroscopy and started applying this technique to investigate a variety of mechanochemically induced organic reactions [6,10,18]. The combination of mechanochemistry and solid-state NMR spectroscopy also allowed us to analyze the proceedings of molecular-recognition events. In our initial study [10], we milled mixtures of enantiomers (artificial conglomerates) of various compounds including the two amino acids serine and alanine in a ball mill and followed the formation of the respective racemic phases by solid-state NMR. It became apparent that, for some compounds, such processes occurred nearly instantaneously, whereas racemic-phase formations of alanine and serine required more intense (ball) milling techniques. The less powerful RAM (for a detailed explanation of the RAM device, please refer to reference [19]) [20,21,22,23,24,25], which can be beneficial for reactions involving compounds that are sensitive to the forces present in a ball mill, remained ineffective for the two amino acids [10]. Realizing, however, that we had not applied optimal RAM conditions and that additives could strongly affect mechanochemical reactions [26,27], we revisited the amino acid systems and started varying the conditions of the RAM. As a result, we identified a number of parameters which, after optimization, led to a robust protocol providing reproducible results that is reported in this work.

## 2. Results and Discussion

### 2.1. Determination of Enantiomeric Excesses from Carbon-13-Detected MAS Spectra

**Figure 1:**
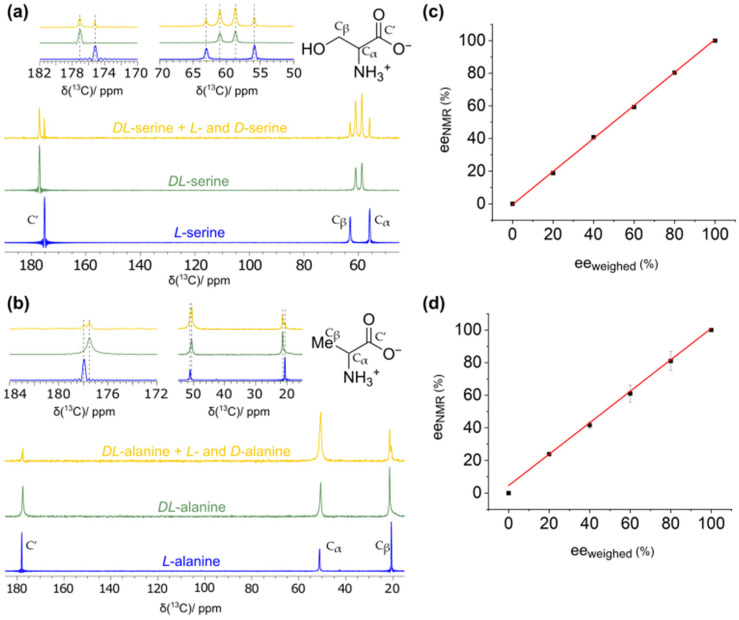
^13^C CP-MAS NMR spectra showing (**a**) differences in chemical-shift values between *L*- and *DL*-serine (blue and green spectrum, respectively) and (**b**) between *L*- and *DL*-alanine (blue and green spectrum, respectively). In addition, the ^13^C spectra of a representative scalemic mixture of *enantiopure* and *DL*-amino acids are shown (yellow; a mixture of the *enantiopure L*- and *D*- amino acids as well as the corresponding *DL*-amino acids formed from them). The truncation in some of the spectra is caused by too short data acquisition times, caused by limitations due to the higher-power proton decoupling. (**c**,**d**) show the correlations between theoretically expected *ee*-values (based on the weighed in amounts of *L*- and *DL*-amino acids) and the *ee*-values determined by NMR for serine and alanine based on the simple integration of the NMR resonances. The red lines correspond to linear regressions with R^2^-values of 0.999 and 0.996 for serine and alanine, respectively. All spectra were recorded at 11.7 T, 285 K and 17.0 kHz MAS frequency.

The unambiguous distinction between enantiopure and racemic phases of the two amino acids, alanine and serine, is central for this study. This can be achieved by ^13^C-detected solid-state NMR spectroscopy due to small chemical-shift differences between the phases [10], which result from small structural differences in their respective single-crystal structures. Figure 1a,b display the ^1^H-^13^C cross-polarization (CP)-MAS NMR spectra under MAS conditions of *L*- and *DL*-serine, as well as *L*- and *DL*-alanine, respectively. And indeed, differences in the ^13^C chemical-shift values, *δ*(^13^C), allow for a straightforward distinction of such phases. As reported previously, the differences are larger for serine than for alanine [10]. Also noteworthy, the enantiopure and racemic phases within scalemic mixtures of *L*-, *D*- and *DL*-amino acids (ratios 75% *DL*-serine to 25% enantiopure serine and 64% *DL*-alanine to 36% enantiopure alanine as representative examples; see yellow spectra in Figure 1a,b) can clearly be differentiated in the ^13^C CP-MAS spectra in both cases.

However, the spectra were recorded with a CP polarization transfer step, which is a priori not quantitative. Following our recent studies on the small organic molecule trifluoromethyl lactic acid (TFLA) [10], we have determined the enantiomeric excesses (*ee*) for a selection of scalemic mixtures of the *L*- and *DL*-amino acids by comparing the ratios of the ^13^C CP-MAS NMR resonances obtained from simple integration to illustrate that, in first approximation, the CP-spectra can directly be used for the *ee*-determination. In that vein, the corresponding “calibration curves” in which the NMR-derived *ee*-value is plotted against the theoretically expected *ee*-value (the latter have been determined based on the weighed in amounts of the *L*- and *DL*-amino acids) is depicted in Figure 1c,d. In both cases, a linear correlation is observed with slopes of 1.01 ± 0.01 and 0.96 ± 0.03 and *y*-axis intercepts at −0.4% and 4.6% for serine and alanine, respectively. We therefore use, in the following, the peak integrals of the ^13^C solid-state NMR spectra as a reasonable estimate for determining *ee*-values without applying any further correction to those values (for more details, see the experimental section in the SI).

### 2.2. Efforts Towards Formation of the Racemic Phase by Resonant Acoustic Mixing

**Figure 2:**
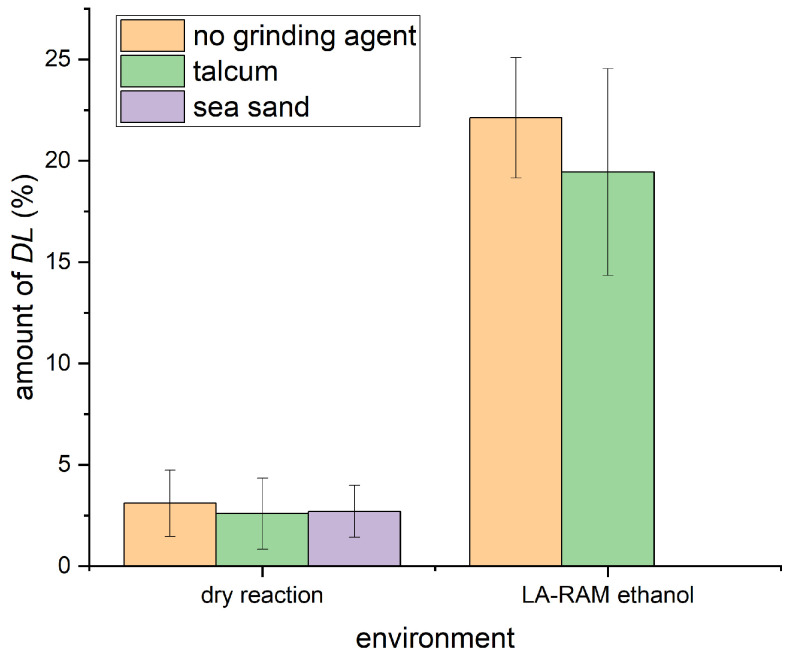
Amount of formed *DL*-serine starting from an equimolar mixture of *D*- and *L*-serine in dry and LA-RAM conditions using ethanol (η=0.2 µL/mg). All samples were processed in the RAM device for 20 min at 100× *g*.

We intended to identify the triggers that could induce racemic-phase formation by using the RAM device. We have previously found that the formation of racemic serine and alanine was possible in a solvent-free environment in the ball mill [10,28,29]. When, however, transitioning these systems to the RAM device, only minimal amounts of racemic phases were formed [30]. We hypothesized that adding grinding agents such as sand and talcum to the reaction mixture could enhance the reactivity of the system. Sand is sometimes used to improve the crushing of materials in the ball mill [31], while talcum potentially acts as a lubricant, preventing the material from sticking to walls and thus in participating further in the reaction [32,33]. Some of us observed positive effects of talcum in mechanochemical metal-catalyzed C–X- and X–Y-bond formations [34,35,36]. In the cases studied here, however, both additives showed no positive effect and the amount of formed *DL*-serine even decreased as shown in Figure 2. While talcum circumvented the sticking of powder to the walls of the vessel, it also resulted in the formation of small round clumps being formed with the material (see Figure S1 in the Supporting Information).

We also tested the addition of small amount*s* of solvents to the reaction to imitate well-established liquid-assisted grinding procedure*s*. Adding solvents has already been used intensively in both milling and RAM (liquid-assisted grinding, LAG, and LA-RAM), and the benefits are highlighted in several reports (for a selection, see [16,22,37,38,39]).

In our case, the initial tests were performed with ethanol (η=0.2 µL/mg). The η-value in LA-RAM describes the amount of μL of solvent for every mg of solid material used [40]. Here, the addition of the liquid significantly increases the amount of racemic phase formed, e.g., by adding ethanol, around 20% of racemic phase was observed for both amino acids investigated (Figure 2) [41]. Again, the presence of talcum led to a slightly smaller amount of the racemic phase.

### 2.3. Formation of the Racemic Phases by LA-RAM

**Figure 3:**
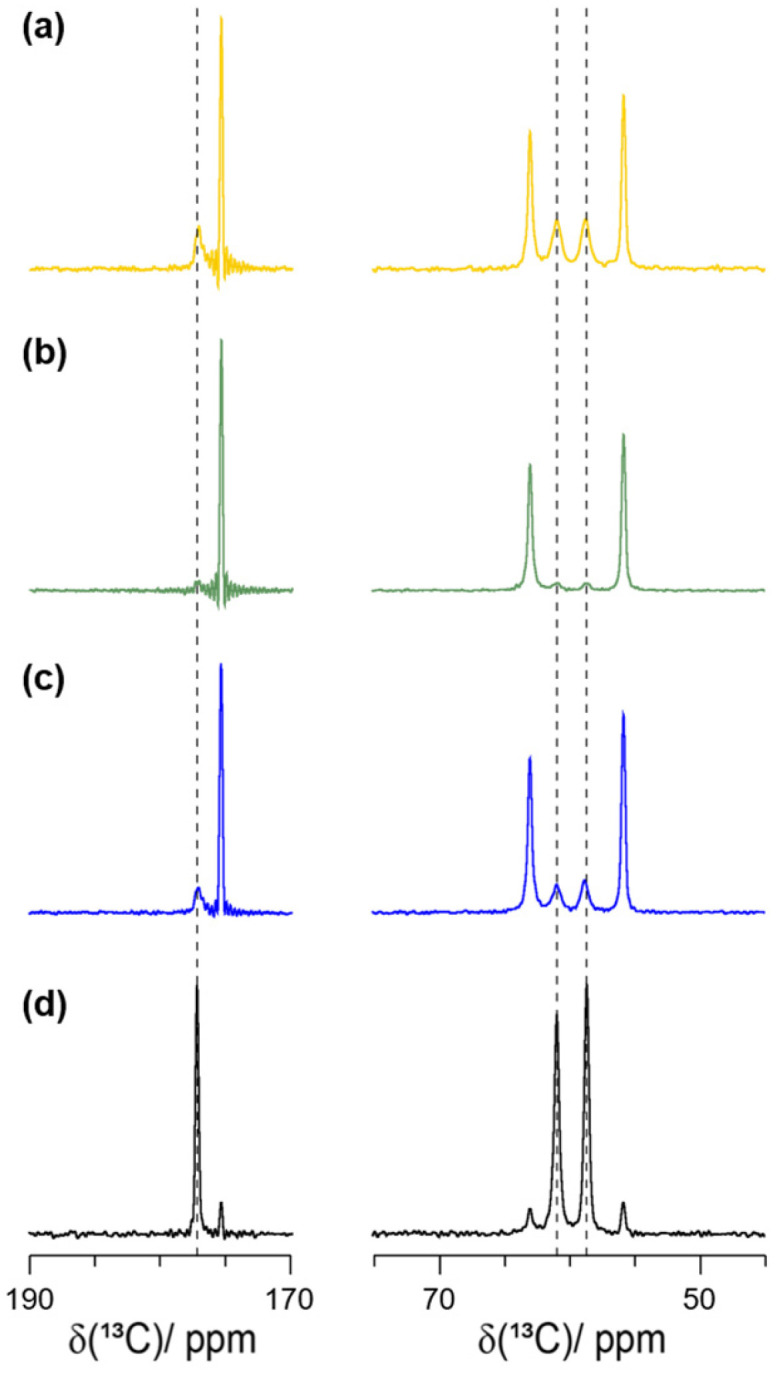
Zoomed-in ^1^H-^13^C CP-MAS spectra of *DL*-serine prepared under LA-RAM condition (η=0.2 µL/mg, 100 g, 20 min) with (**a**) DMSO, (**b**) acetonitrile, (**c**) ethanol, and (**d**) de-ionized water as the solvent. All spectra were recorded at 11.7 T, 285 K and 17.0 kHz MAS frequency. Dashed lines highlight *DL*-serine resonances. The truncation in some of the spectra is caused by too short data acquisition times, caused by limitations due to the higher-power proton decoupling.

Encouraged by the initial LA-RAM results using ethanol, we next varied the solvent used. The previously applied conditions in the ball mill were imitated as closely as possible by using the same amount of material, an η-value of 0.2 µL/mg, a processing time of 20 min, and applying the highest possible energy input of the RAM device, which is 100× *g*. Note that, as outlined in previous work [10], we did not follow optimal RAM operation parameters (e.g., with respect to the filling factor of the vials) due to chemical restraints. We tested multiple solvents with different polarities for their efficacy in racemic-phase formation. Excerpts of the ^13^C CP-MAS NMR spectra of serine are shown in Figure 3. The complete spectra for serine and alanine can be found in Figures S2 and S3.

**Figure 4:**
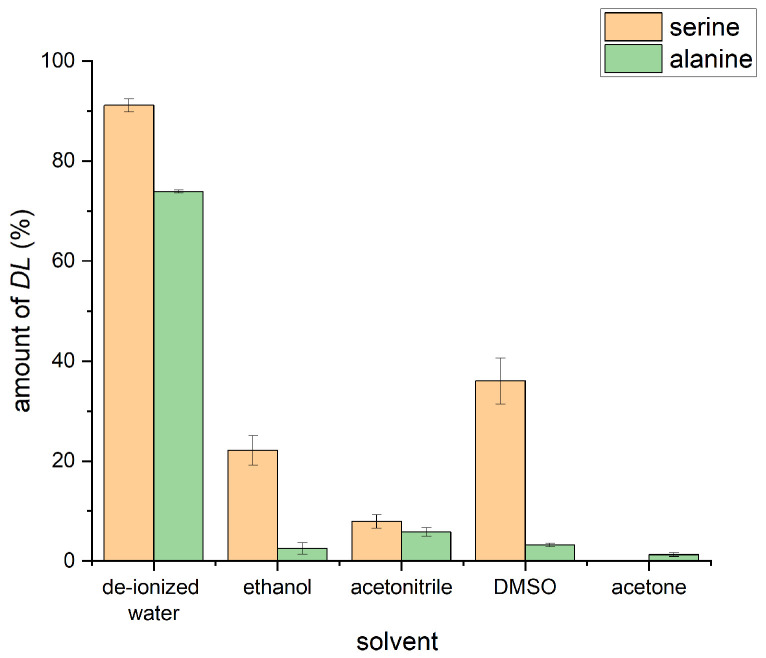
Amount of racemic amino acid phase formed in LA-RAM upon variation of the solvent used (η=0.2 µL/mg). Data for alanine and serine are shown. The samples were processed in the RAM device for 20 min at 100× *g*.

The amount of racemic phase formed in each case is reported in Figure 4. For both amino acids, de-ionized water led to the highest amount of the racemic phase. The results also reveal clear differences between serine and alanine for the used solvents. We hypothesize that, at least in part, the efficiency of *DL*-serine formation is related to the solubility in the explored solvents, with the highest solubility for serine in water followed by acetonitrile and ethanol. Alanine shows the highest solubility in water, followed by ethanol and by acetonitrile [42,43]. Both serine and alanine are insoluble (<1 mg/mL) in DMSO [44,45]. As Figure 4 reveals, our experimental data do not entirely follow these solubility trends, pointing to a more complex interplay of the solvent properties on the solid-state molecular-recognition process studied herein.

### 2.4. η-Optimization

**Figure 5:**
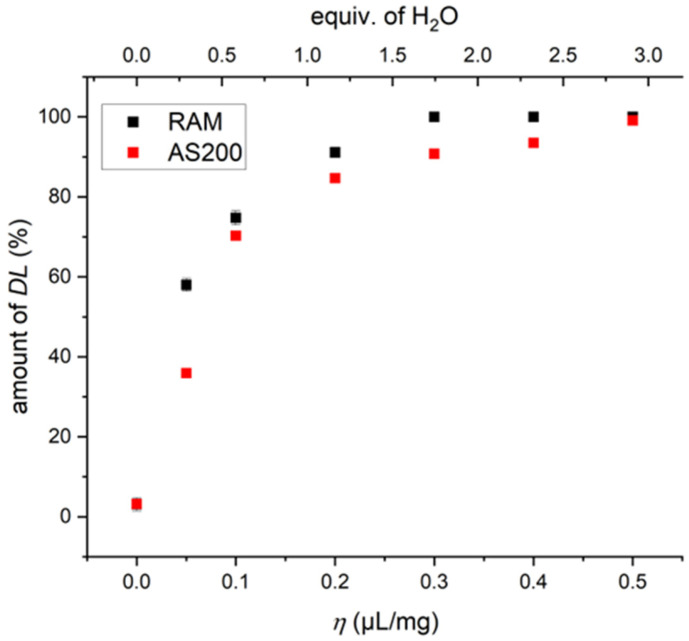
*DL*-serine formation in the RAM device and the AS200 as a function of the η-parameter. De-ionized water was used as solvent. The samples were processed in the RAM device for 20 min at 100× *g* (black data points) or in the AS200 for 20 min at 25× *g* (red data points).

Next, we optimized the η-value for the racemic-phase formation using de-ionized water. Six samples with η-values ranging from 0.05 µL/mg to 0.5 µL/mg were prepared and studied by ^13^C-detected solid-state NMR. As discussed in more detail below, these investigations were then extended by using an AS200 basic vibratory sieve shaker provided by RETSCH (Haan, Germany) instead of the RAM device. In Figure 5, the amount of the racemic phase of serine formed in both approaches is plotted against the η-value as well as the equivalents of water. (See Figures S4 and S5 as well as Tables S1 and S2 in the Supporting Information for the spectra and values, respectively)

The results show that even the lowest amount of water added (0.05 µL/mg) already led to a significant amount of racemic phase (~50%). In the case of serine, it was possible to reach 100% *DL*-serine with η=0.3 µL/mg. Notably, such an *η*-value, although small, already corresponds to an excess of water with respect to a serine monomer, which suggests a special role of water in our studies (vide infra) [46].

As noted before, the *DL*-serine phase formation was also studied in the AS200 basic vibratory sieve shaker. This device is based on a similar movement pattern as the RAM device with the difference that it includes a small circular shift. Thus, the same experiments with serine as performed in the RAM device were repeated with the AS200. In this case, however, only 25× *g* was applied (for 20 min) as that was the maximum possible acceleration for the device. The trend for *DL*-serine formation as a function of the η-value in the AS200 was rather similar to that of the RAM device. The overall slightly lower efficiency was attributed to the lower acceleration compared to the RAM device. The ^13^C CP-MAS NMR spectra reveal in some spectra minor resonances from *L*- or *D*-serine monohydrate. Besides these differences, the AS200 appeared to be a viable alternative to the RAM device when performing these kinds of reactions.

In contrast to serine, the amount of *DL*-alanine increased slightly slower as a function of η and only reached around ~95% *DL*-alanine with η=0.5 µL/mg (for further details, see Figures S6, S7 and Table S3). With these η-values, it was possible to push the processing time down to one minute in the RAM device with approximately 99% *DL*-serine and approximately 95% *DL*-alanine formed, illustrating the extremely efficient molecular-recognition processes. The η-correlations observed herein are different from those reported for a mechanoredox diazonium borylation [21] or for a Suzuki coupling reaction [16]. In both cases, the reaction yield dropped for higher η-values after a maximum was reached, which is not observed in our cases, at least for the range of η-values studied. We again hypothesize that, in our case, water plays a special role (vide infra).

### 2.5. The Influence of Water on Different Serine Phases

**Figure 6:**
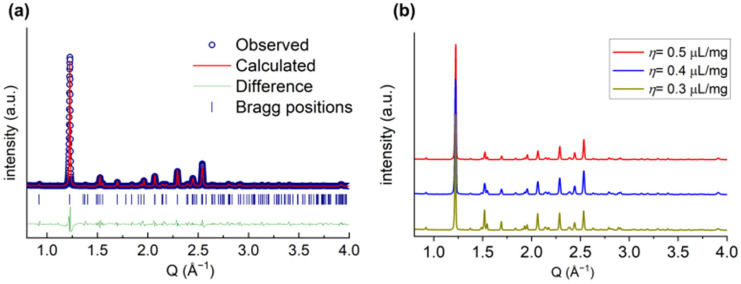
(**a**) Rietveld refinement of PXRD measurement of a *DL*-serine sample for η=0.5 µL/mg prepared in the RAM device. (**b**) PXRD patterns of *DL*-serine with different *η*-values, stacked in offset for clarity.

The phase purity of the *DL*-amino acids formed in the RAM device was further cross-validated by powder X-ray diffraction (PXRD) experiments. All diffraction patterns could be modeled with the space group P2_1_/a, characteristic of the *DL*-serine crystal structure [47], with marginal variations in unit cell parameters for the different *η*-values used (see Table S4). An example of the refinement for the sample with η=0.5 µL/mg is shown in Figure 6a, while a comparison of the different diffraction patterns for samples with different *η*-values is shown in Figure 6b. A refinement of a *L*-serine sample as a comparison is shown in Figure S8. The differences between the refinements in Figure 6a and Figure S8 further strengthen our observations of a phase transition to the *DL*-amino acids in our experiments. The cell parameters obtained from the diffraction patterns are compiled in Table S4.

**Figure 7:**
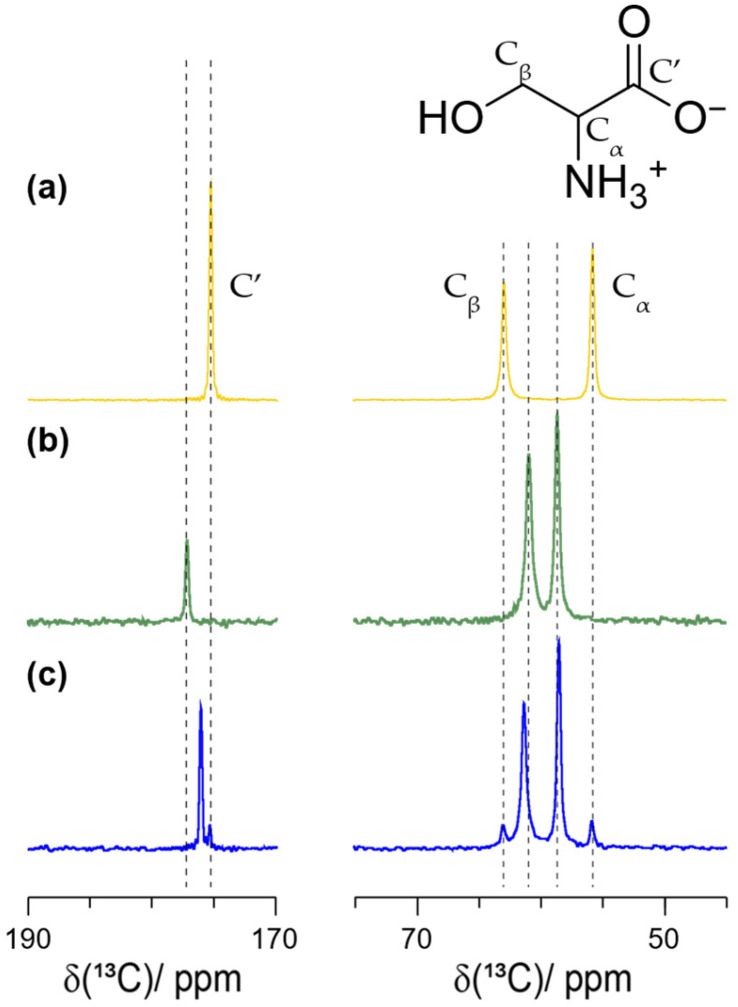
^1^H-^13^C CP-MAS NMR spectra of (**a**) purchased *L*-serine (yellow) as well as (**b**) purchased *DL*-serine (green) and (**c**) *L*-serine monohydrate formed in the RAM device (blue). The less intense resonances in the *L*-serine monohydrate spectrum point to a residual amount of anhydrous *L*-serine in the sample. Dashed lines highlight *L*- and *DL*-serine resonances, respectively. All spectra were recorded at 11.7 T, 285 K and 17.0 kHz MAS frequency.

We also subjected *L*-serine in the absence of the second enantiomer to LA-RAM (η=0.3 µL/mg). This led to the formation of the monohydrate serine phase which is unstable under MAS conditions as reported previously [48] (see Figure S9 for time-dependent spectral changes). The corresponding ^13^C CP-MAS NMR spectrum is shown in Figure 7. It has rather similar Cα and Cβ chemical-shift values than the *DL*-serine phase. However, a clear chemical-shift difference is observed for the carbon of the carboxylic acid group, still allowing the unambiguous spectroscopic distinction of the two phases. The formation of the monohydrate phase has also been proven by PXRD (see Figure S10).

**Figure 8:**
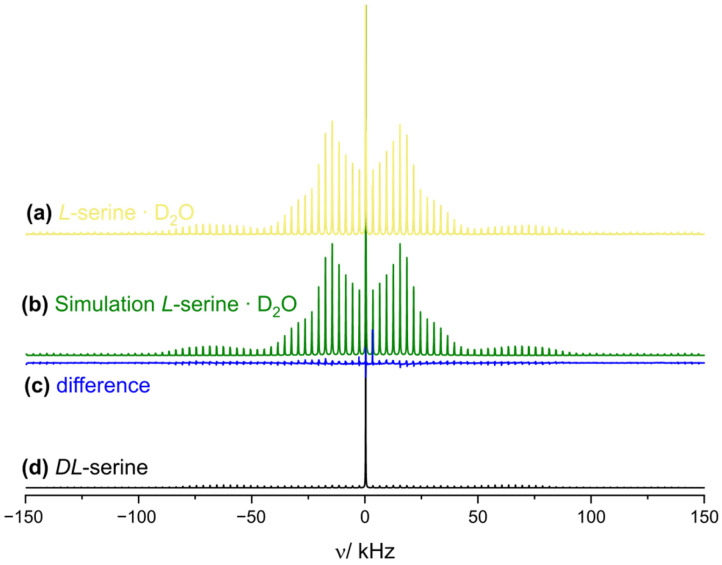
^2^D MAS spectra of *L*-serine ∙ D_2_O (yellow) with a line shape simulation (green) of the *L*-serine ∙ D_2_O spectrum and the difference (blue) between them as well as *DL*-serine (black) prepared by RAM. All spectra were recorded at 11.7 T, 285 K and 3.0 kHz MAS frequency.

To further corroborate the PXRD data pointing to the absence of water in the *DL*-serine phase, *DL*-serine was prepared in the RAM device using D_2_O as the solvent. As a reference, the *L*-serine ∙ D_2_O phase was also synthesized in the RAM device. Deuterium MAS NMR spectra (^2^D possesses a nuclear spin quantum number of *I* = 1) are highly sensitive for molecular motion [49]. In the case of crystal hydrates, the first-order quadrupolar coupling interaction causes an MAS spinning sideband pattern in slow-spinning MAS experiments whose envelope is a symmetric doublet powder line shape that encodes for the quadrupolar coupling constant and thus the underlying molecular dynamics [50,51,52]. Entirely rigid D_2_O molecules lead to quadrupolar coupling constants (C_Q_) of around 210 kHz and asymmetry parameters (η_Q_) close to zero (values for ice) [50], whereas local motions (typically rapid flips along the C_2_-symmetry axis) reduce the observed C_Q_-values and lead to increased η_Q_-values [50,51]. And indeed, a first-order quadrupolar sideband pattern is observed in the ^2^D MAS NMR spectrum for the *L*-serine ∙ D_2_O phase (Figure 8a), wherein all exchangeable protons have been (partially) substituted by deuterons. The spectrum can be simulated assuming four resonances, namely ammonium deuterons (δ_iso_ = 7.6 ppm, C_Q_ = 53 kHz and η_Q_ = 0.25), hydroxyl deuterons (δ_iso_ = 7.5 ppm, C_Q_ = 210 kHz and η_Q_ = 0.13), rigidified crystal D_2_O (δ_iso_ = 5.1 ppm, C_Q_ = 208 kHz and η_Q_ = 0.14), as well as fully mobile D_2_O adsorbed on the surface of the crystalline material (δ_iso_ = 4.9 ppm) (for the simulation, see Figure 8b; for more details, see Figure S11). Such values agree with reported ones [53,54] and clearly reveal the presence of (relatively rigid bound) crystal water in the *L*-serine monohydrate phase. In contrast, the ^2^D MAS NMR spectrum of *DL*-serine prepared in the RAM device shows an intense sharp resonance with a single MAS sideband pointing to rather freely tumbling D_2_O molecules probably adsorbed on the surface of the formed *DL*-serine crystalline phase. In addition, a weak sideband pattern caused by deuterated hydroxyl protons and deuterated amino protons is visible, indicating minor hydrogen to deuterium exchange prior to racemic-phase formation (Figure 8c and Figure S12). This supports the PXRD data showing the formation of anhydrous *DL*-serine. Purchased *DL*-serine is less prone to deuteron exchange in the RAM device than the enantiopure phase, as concluded from the less intense deuterium sideband pattern observed for purchased *DL*-serine subjected to RAM in presence of D_2_O; this is most likely caused by its lower solubility in water compared to its enantiopure counterpart, as concluded from the less intense deuterium sideband pattern (Figure S13) [55]. We thus hypothesize that the formation of *DL*-serine in the RAM device occurs in a similar timeframe as the deuteron exchange in the enantiopure entities takes place. In conclusion, we imagine that RAM could become a versatile tool for the deuteration of exchangeable protons in organic solids.

Finally, we noted that the formation of the racemic phase in the presence of water occurs rather easily, even by storing a mixture of *L*- and *D*-serine for three days in a desiccator with a humidity of >95%. In this case, full conversion of the enantiopure phases into the racemic phase was observed. Repeating the experiment by bringing *L*- and *D*-serine only into contact (no mixing was applied), we observed that the racemic phase was only formed at the contact surface. In the areas outside the contact surface, enantiopure and/or the corresponding monohydrate phases were found (see Figure S14). These observations are in line with those described in a previous report on the importance of humidification in racemic serine formation [28,46].

## 3. Materials and Methods

### 3.1. Chemicals

All chemicals used were obtained from commercial sources. Serine and alanine were first processed in the ball mill with the goal to increase their surface area by reducing particle sizes. Batches of 1 g of serine or alanine were treated for 20 min at a frequency of 25 Hz in a 10 mL stainless steel jar with one 10 mm stainless steel milling ball. The solvents were used without further purification. Further information about the chemicals is listed in Table 1 and Table 2.

### 3.2. Equipment

A MM400 shaker ball mill (RETSCH, Haan, Germany) was used to process the chemicals before the experiments. For that, 10 mL stainless steel jars in combination with a 10 mm stainless steel milling ball were used. Racemic-phase formation was performed using the *Lab*RAM I from Resodyn Acoustic Mixers (Butte, MT, USA) in 2 mL snap cap vials in a custom vial carrier (see Figure S15) [19,56].

The NMR experiments were performed in 3.2 mm zirconia rotors with vespel caps (Bruker Biospin) using a 3.2 mm triple-resonance HXY probe in a 500 MHz Bruker NMR-spectrometer (Avance 3 HD console). Further information regarding the NMR measurements can be found in Table S5. Further detailed information about the work procedures can also be found in the Supporting Information.

The powder X-ray diffraction (PXRD) patterns were collected with a D8 Advance diffractometer (Bruker, Berlin, Germany) with Cu Kα radiation (λ = 1.5406 Å) in the momentum transfer (Q) range 0.7–4.1 Å^−1^. Structure refinements with the Rietveld method were performed using GSAS-II software (version 5609) [57].

## 4. Conclusions

In conclusion, we explored the potential of RAM in solid-state molecular-recognition events using the examples of the amino acids alanine and serine. It was shown that LA-RAM can be used to efficiently form the racemic phases of both, alanine and serine, by mixing equimolar amounts of the enantiopure entities. Water proved to be the solvent of choice for LA-RAM. Similar results were obtained when an AS200 basic vibratory sieve shaker was used instead of the RAM device. ^13^C-detected solid-state NMR served as a valuable tool to monitor the racemic-phase formation. RAM can also be used for the deuteration of organic solids. We strive to gain a better understanding of molecular recognition in solids and as such a better insight into mechanochemical processes in general. This shall be beneficial for a multitude of research fields ranging from organic synthesis of pharmaceutically relevant molecules, in particular chiral ones, where resolutions of racemates can give enantiopure products, to materials where preparative aspects and degradation processes are important for recycling.

## Figures and Tables

**Table 1:** List of used amino acids.

Chemical	CAS Number	Purity	Manufacturer
*L*-serine	56-45-1	99%	abcr GmbH (Karlsruhe, Germany)
*D*-serine	312-84-5	98%	abcr GmbH (Karlsruhe, Germany)
*DL*-serine	302-84-1	99%	abcr GmbH (Karlsruhe, Germany)
*L*-alanine	56-41-7	-	Degussa (Wesseling, Germany)
*D*-alanine	338-69-2	-	Degussa (Wesseling, Germany)
*DL*-alanine	302-72-7	-	-

**Table 2:** List of used solvents.

Solvent	CAS Number	Purity	Manufacturer
Water	-	De-ionized	-
Ethanol	64-17-5	Technical grade	Julius Hoesch GmbH & Co. KG (Düren, Germany)
Acetonitrile	75-05-8	>99.9%	Riedel-de Haën (Seelze, Germany)
Dimethyl sulfoxide (DMSO)	67-68-5	99.7%	Thermo scientific (Waltham, MA, USA)
Deuterium oxide	7789-20-0	Analytical grade	Eckert & Ziegler Chemotrade GmbH (Berlin, Germany)

## Data Availability

Data are contained within the article or Supplementary Materials. Additional datasets are available on request from the authors.

## Supplementary Materials

The following supporting information can be downloaded at https://www.mdpi.com/article/10.3390/molecules30183745/s1, Figure S1. Clumped serine material obtained after the addition of talcum powder in the resonant acoustic mixer. For this experiment, 25 mg of *L*-serine was combined with 25 mg of *D*-serine and 25 mg of talcum powder in a 2 mL snap cap vial. It was then processed in the resonant acoustic mixer for 20 min at 100× *g*; Figure S2. ^1^H-^13^C CP-MAS spectra of *DL*-serine prepared under LA-RAM conditions (η=0.2 µL/mg, 100× *g*, 20 min) with (a) DMSO, (b) acetonitrile, (c) ethanol and (d) deionized water as the solvent. All spectra were recorded at 11.7 T, 285 K and 17.0 kHz MAS frequency. Dashed lines highlight *DL*-serine resonances. The truncation in some of the spectra is caused by too short data acquisition times; Figure S3. ^1^H-^13^C CP-MAS spectra of *DL*-alanine prepared under LA-RAM conditions (η=0.2 µL/mg) with (a) DMSO, (b) acetonitrile, (c) ethanol and (d) deionized water as the solvent. All spectra were recorded at 11.7 T, 285 K and 17.0 kHz MAS frequency. Dashed lines represent *DL*-alanine resonances. The lower signal-to-noise ratio in some of the spectra is caused by a less efficient CP polarization transfer probably due to the partial dissolution of the sample in the solvent used; Figure S4. ^1^H-^13^C CP-MAS spectra of *DL*-serine prepared under LA-RAM conditions (deionized water, 100× *g*, 20 min) with (a) η=0 µL/mg, (b) η=0.05 µL/mg, (c) η=0.1 µL/mg (d) η=0.2 µL/mg (e) η=0.3 µL/mg (f) η=0.4 µL/mg and (g) η=0.5 µL/mg. All spectra were recorded at 11.7 T, 285 K and 17.0 kHz MAS frequency. The truncation in some of the spectra is caused by too short data acquisition times; Table S1. The amounts of formed *DL*-serine calculated from the spectra in Figure S4 in relation to the η-value of deionized water used; Figure S5. ^1^H-^13^C CP-MAS spectra of *DL*-serine prepared under AS200 conditions (deionized water, 25× *g*, 20 min) with (a) η=0 µL/mg, (b) η=0.05 µL/mg, (c) η=0.1 µL/mg (d) η=0.2 µL/mg (e) η=0.3 µL/mg (f) η=0.4 µL/mg and (g) η=0.5 µL/mg. All spectra were recorded at 11.7 T, 285 K and 17.0 kHz MAS frequency. The truncation in some of the spectra is caused by too short data acquisition times. In some spectra, the monohydrate phase is observed in very small amounts (black arrows); Table S2. The amounts of formed *DL*-serine calculated from the spectra in Figure S5 in relation to the η-value of deionized water used; Figure S6. *DL*-alanine formation in the resonant acoustic mixer as a function of η-parameter. Deionized water was used as solvent. The samples were processed in the resonant acoustic mixer for 20 min at 100× *g*; Figure S7. ^1^H-^13^C CP-MAS spectra of *DL*-alanine prepared under LA-RAM conditions (deionized water, 100× *g*, 20 min) with (a) η=0 µL/mg, (b) η=0.05 µL/mg, (c) η=0.1 µL/mg (d) η=0.2 µL/mg (e) η=0.3 µL/mg (f) η=0.4 µL/mg and (g) η=0.5 µL/mg. All spectra were recorded at 11.7 T, 285 K and 17.0 kHz MAS frequency. The truncation in some of the spectra is caused by too short data acquisition times. The lower signal-to-noise ratio in some of the spectra is most likely caused by the partial dissolution of the sample in the solvent used rendering the CP polarization transfer less efficient; Table S3. The amounts of *DL*-alanine calculated from the spectra in Figure S7 in relation to the η-value of deionized water used; Table S4. Results of Rietveld refinement of PXRD data presented in Figure 6 of the main text; Figure S8. Rietveld refinement of PXRD measurement of *L*-serine samples. The deviations from the fit can be attributed to the strong preferred orientation of serine as well as the influence of air moisture on the sample; Figure S9. The correlation between the amount of *L*-serine monohydrate present in the sample over time. The error bars are calculated as the standard deviation of the amounts of racemic amino acid gained from the integration of the NMR signals. The error bars are calculated as the standard deviation of the amounts of racemic amino acid gained from the integration of the NMR signals; Figure S10. Rietveld refinement of PXRD measurement of *L*-serine monohydrate samples prepared in the resonant acoustic mixer. The deviations from the fit can be attributed to the strong preferred orientation of serine as well as the sample reverting to the anhydrous serine phase over time during the measurement; Figure S11. ^1^H-^13^C CP-MAS spectra of purchased *L*-serine (yellow) as well as purchased *DL*-serine (green) and *L*-serine monohydrate formed in the resonant acoustic mixer (blue). The less intense resonances in the *L*-serine monohydrate spectrum (blue) point to a residual amount of water-free *L*-serine in the sample. All spectra were recorded at 11.7 T, 285 K and 17.0 kHz MAS frequency; Figure S12. ^2^D MAS spectrum (black) and simulations of *L*-serine monohydrate. The spectrum was recorded at 11.7 T, 285 K and 3.0 kHz MAS frequency. The simulations were performed using DMFit [58]. Individual contributions to the line shape are plotted as colored lines, the purple line represents the difference between the experimental and simulated spectrum; Figure S13. ^2^D MAS spectrum of *DL*-serine created by combining *D*- and *L*-serine with D_2_O in the resonant acoustic mixer (black) and *DL*-serine processed with D_2_O in the resonant acoustic mixer (blue). The spectra were recorded at 11.7 T, 285 K and 3.0 kHz MAS frequency. The spectra mostly reveal freely tumbling D_2_O molecules adsorbed on the material; Figure S14. ^1^H-^13^C CP-MAS spectra of (a) *L*- and *D*-serine mixed with a spatula, (b) *L*-serine monohydrate on the left side of the contact surface (c) material from the contact surface between *L*- and *D*-serine and (d) *D*-serine monohydrate and anhydrous *D*-serine from the right side of the contact surface. The samples were stored in a desiccator at >95% humidity for approximately 3 days. All spectra were recorded at 11.7 T, 285 K and 17.0 kHz MAS frequency. Dashed lines highlight *DL*-serine resonances. The expected monohydrate phase of *D*-serine in sample (d) has mostly transformed back into its anhydrous phase due to the time delay between taking the entire sample out of the desiccator and the start of measurement; Figure S15. Images of (a) the MM400 shaker mill with its milling vessels to the right and (b) the LabRAM I with the custom-made vial carrier to its right; Figure S16. Images of the AS200 basic vibratory sieve shaker with the sample holder clamped in between the vibration plate and a sample pan; Table S5. Experimental parameters of the conducted ^1^H-^13^C CP MAS experiments; Subsection work procedures.

## Abbreviations

The following abbreviations are used in this manuscript:

CP	Cross-Polarization
LA-RAM	Liquid-Assisted Resonant Acoustic Mixing
MAS	Magic-Angle Spinning
NMR	Nuclear Magnetic Resonance
RAM	Resonant Acoustic Mixing
