# Supplementary material for: Formation of Racemic Phases of Amino Acids by Liquid-Assisted Resonant Acoustic Mixing Monitored by Solid-State NMR Spectroscopy
Source: Molecules. 2025 Sep 15;30(18):3745. doi: 10.3390/molecules30183745 (PMC12472410; doi:10.3390/molecules30183745)
Supplement: Supplementary file 1 [file molecules-30-03745-s001.zip › molecules-3838934-supplementary.pdf]

Supporting information for

Formation of racemic phases of amino acids by liquid-assisted resonant acoustic mixing monitored by solid-state NMR spectroscopy

Leeroy Hendrickx [1,2], Calogero Quaranta [3], Emilian Fuchs [1], Maksim Plekhanov[4], Mirijam Zobel[4], Carsten Bolm* [3], and Thomas Wiegand* [1,2]

[1] Max Planck Institute for Chemical Energy Conversion, Stiftstr. 34-36, 45470 Mülheim/Ruhr, Germany
[2] Institute of Technical and Macromolecular Chemistry, RWTH Aachen University, Worringerweg 2, 52074 Aachen, Germany
[3] Institute of Organic Chemistry, RWTH Aachen University, Landoltweg 1, 52074 Aachen, Germany
[4] Institute of Crystallography, RWTH Aachen University, Jägerstraße 17-19, 52066 Aachen, Germany
* Correspondence: CB Carsten.Bolm@oc.rwth-aachen.de, TW thomas.wiegand@cec.mpg.de;

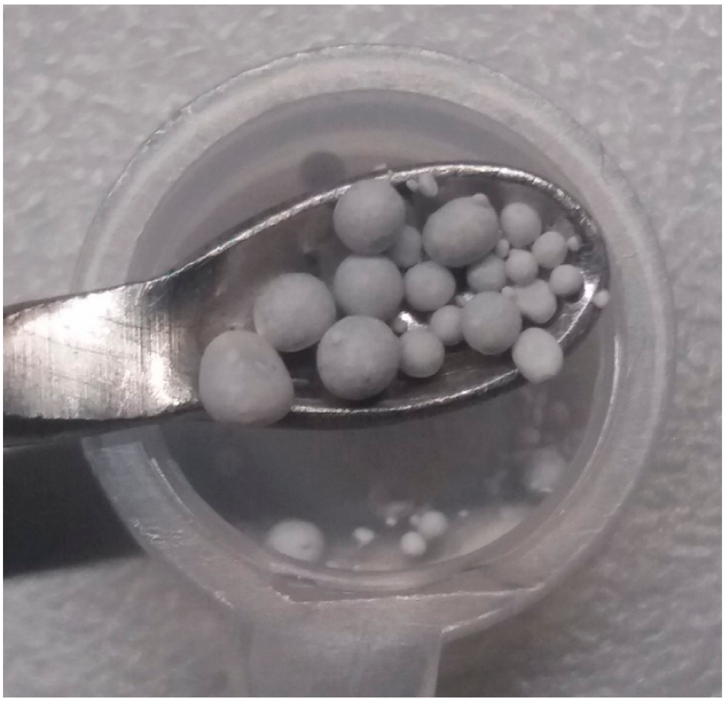

Figure S1. Clumped serine material obtained after the addition of talcum powder in the resonant acoustic mixer. For this experiment, 25 mg of *L*-serine was combined with 25 mg of *D*-serine and 25 mg of talcum powder in a 2 mL snap cap vial. It was then processed in the resonant acoustic mixer for 20 minutes at 100 *g*.

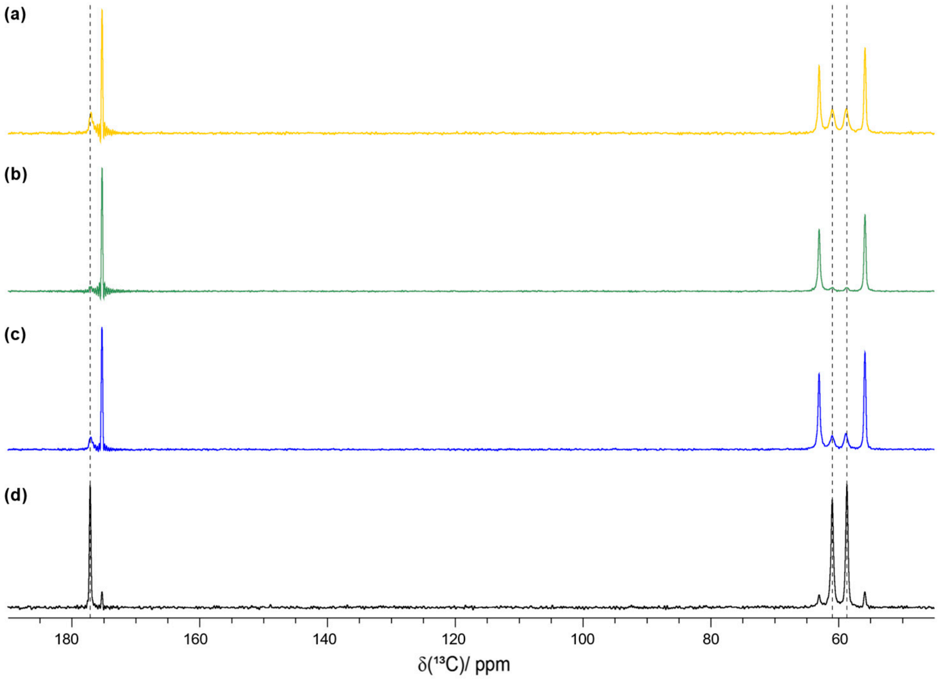

Figure S2. ^{1}H-^{13}C CP-MAS spectra of *DL*-serine prepared under LA-RAM conditions (η = 0.2, 100 *g*, 20 minutes) with (a) DMSO, (b) acetonitrile, (c) ethanol, and (d) deionized water as the solvent. All spectra were recorded at 11.7 T, 285 K and 17.0 kHz MAS frequency. Dashed lines highlight *DL*-serine resonances. The truncation in some of the spectra is caused by too short data acquisition times.

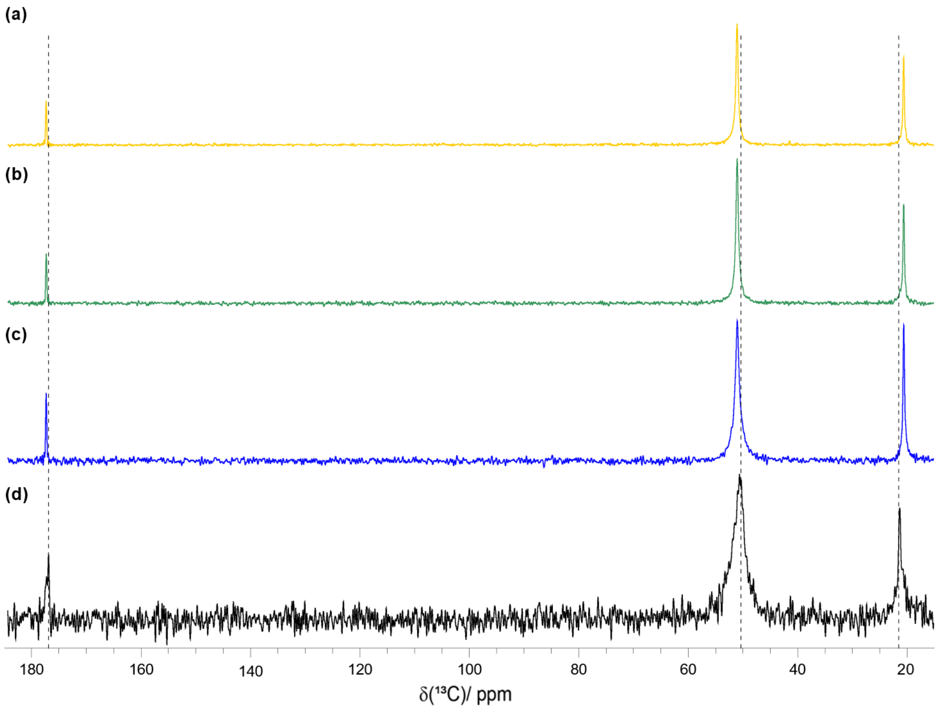

Figure S3. ^{1}H-^{13}C CP-MAS spectra of *DL*-alanine prepared under LA-RAM conditions (η = 0.2, 100 *g*) with (a) DMSO, (b) acetonitrile, (c) ethanol, and (d) deionized water as the solvent. All spectra were recorded at 11.7 T, 285 K and 17.0 kHz MAS frequency. Dashed lines represent *DL*-alanine resonances. The lower signal-to-noise ratio in some of the spectra is caused by a less efficient CP polarization transfer probably due to the partial dissolution of the sample in the solvent used.

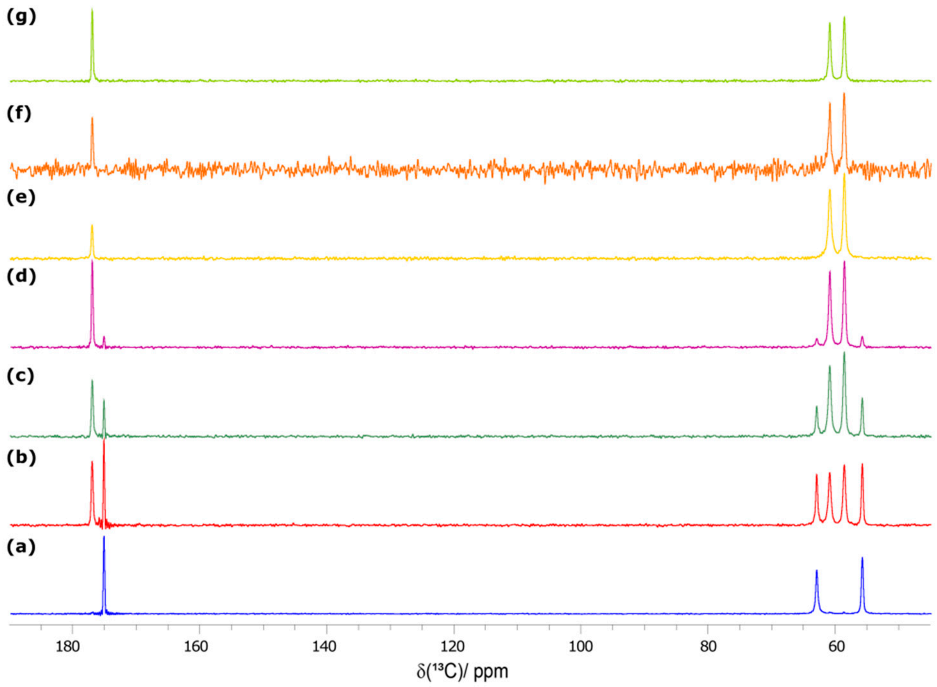

Figure S4. ^{1}H-^{13}C CP-MAS spectra of *DL*-serine prepared under LA-RAM conditions (deionized water, 100 *g*, 20 minutes) with (a) η = 0, (b) η = 0.05, (c) η = 0.1, (d) η = 0.2, (e) η = 0.3, (f) η = 0.4, and (g) η = 0.5. All spectra were recorded at 11.7 T, 285 K and 17.0 kHz MAS frequency. The truncation in some of the spectra is caused by too short data acquisition times.

Table S1. The amounts of formed *DL*-serine calculated from the spectra in Figure S4 in relation to the η-value used.

***η*-value**	**amount of *DL*-Serine [%]**	**standard deviation [%}**
0	3.1	1.6
0.05	58.0	1.6
0.1	74.8	1.8
0.2	91.1	1.3
0.3	100	0
0.4	100	0
0.5	100	0

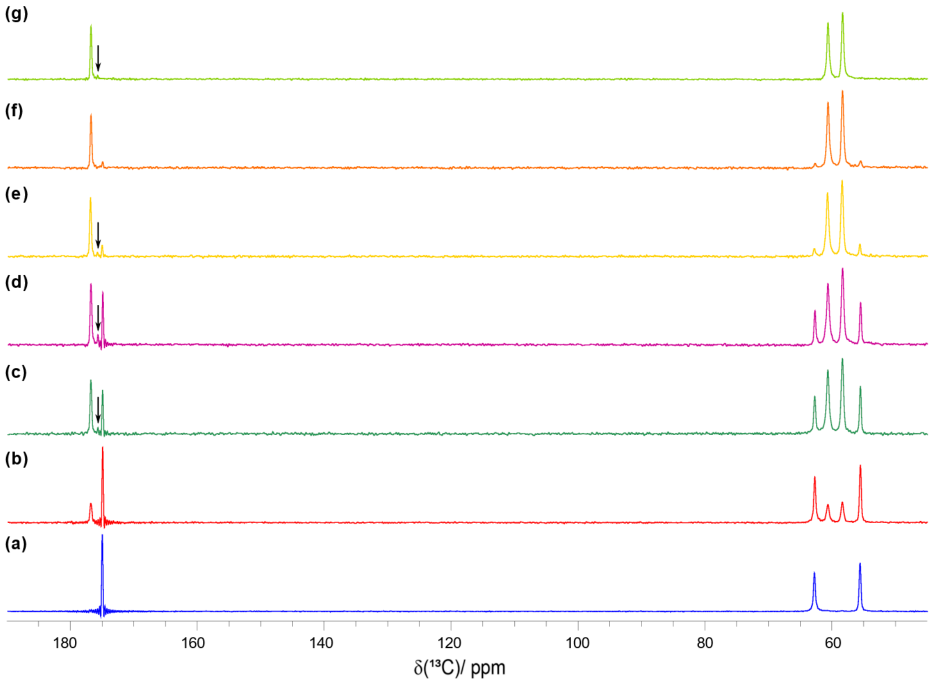

Figure S5. ^1H-^{13}C CP-MAS spectra of *DL*-serine prepared under AS200 conditions (deionized water, 25 *g*, 20 minutes) with (a) η = 0, (b) η = 0.05, (c) η = 0.1, (d) η = 0.2, (e) η = 0.3, (f) η = 0.4, and (g) η = 0.5. All spectra were recorded at 11.7 T, 285 K and 17.0 kHz MAS frequency. The truncation in some of the spectra is caused by too short data acquisition times. In some spectra, the monohydrate phase is observed in very small amounts (black arrows).

Table S2. The amounts of formed *DL*-serine calculated from the spectra in Figure S5 in relation to the η-value used.

**η-value**	**amount of *DL*-Serine [%]**	**standard deviation [%}**
0	3.2	2.5
0.05	35.9	3.4
0.1	70.3	2.9
0.2	84.7	1.0
0.3	90.8	1.1
0.4	93.5	1.8
0.5	99.1	1.7

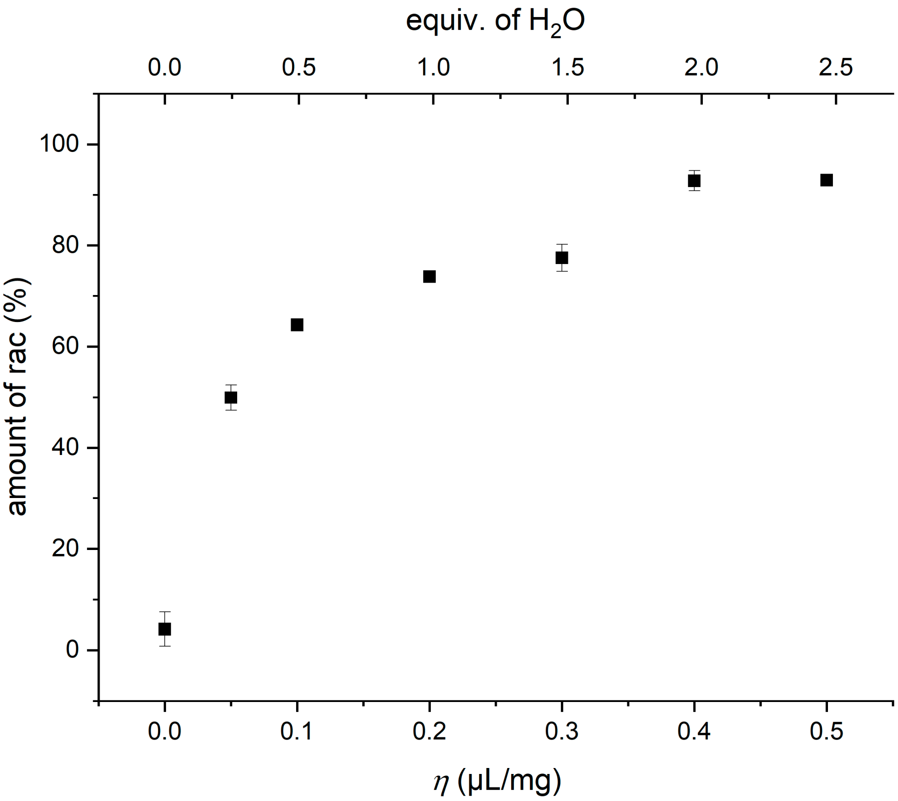

Figure S6. *DL*-alanine formation in the resonant acoustic mixer as a function of η-parameter. Deionized water was used as solvent. The samples were processed in the resonant acoustic mixer for 20 minutes at 100 *g*.

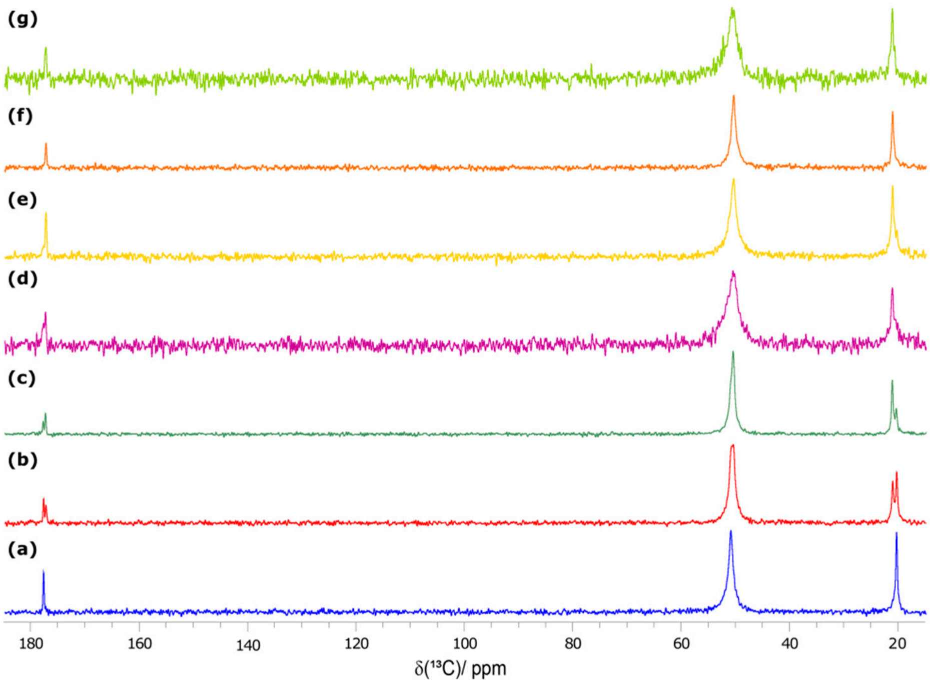

Figure S7. ^{1}H-^{13}C CP-MAS spectra of *DL*-alanine prepared under LA-RAM conditions (deionized water, 100 *g*, 20 minutes) with (a) $\eta = 0$, (b) $\eta = 0.05$, (c) $\eta = 0.1$, (d) $\eta = 0.2$, (e) $\eta = 0.3$, (f) $\eta = 0.4$, and (g) $\eta = 0.5$. All spectra were recorded at 11.7 T, 285 K and 17.0 kHz MAS frequency. The lower signal-to-noise ratio in some of the spectra is most likely caused by the partial dissolution of the sample in the solvent used rendering the CP polarization transfer less efficient.

Table S3. The amounts of *DL*-alanine calculated from the spectra in Figure S7 in relation to the η-value used.

**η-value**	**amount of *DL*-alanine [%]**	**standard deviation [%}**
0	4.2	3.4
0.05	49.9	2.5
0.1	64.3	0.7
0.2	73.9	0.3
0.3	77.6	2.7
0.4	92.8	2.0
0.5	93.0	0.4

Table S4. Results of Rietveld refinement of PXRD data presented in Figure 6 of the main text.

Sample η-value [μL/mg]	*Parameter*	Value
0.5	Space group	$P2_1/a$
	a	10.700 ± 0.003 Å
	b	9.153 ± 0.001 Å
	c	4.817 ± 0.001 Å
	β	106.410 ± 0.005°
	R_w	22.1 %
0.4	Space group	$P2_1/a$
	a	10.717 ± 0.003 Å
	b	9.141 ± 0.001 Å
	c	4.823 ± 0.001 Å
	β	106.457 ± 0.005°
	R_w	19.9 %
0.3	Space group	$P2_1/a$
	a	10.723 ± 0.002 Å
	b	9.145 ± 0.001 Å
	c	4.828 ± 0.001 Å
	β	106.460 ± 0.003°
	R_w	15.5 %

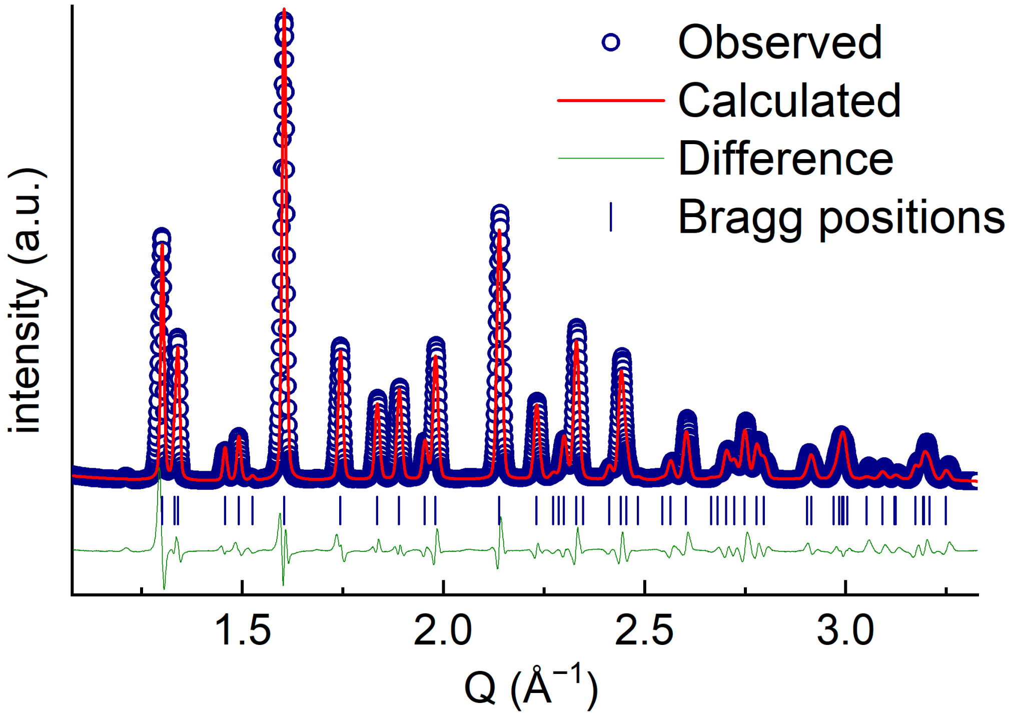

Figure S8: Rietveld refinement of PXRD measurement of *L*-serine samples. The deviations from the fit can be attributed to the strong preferred orientation of serine as well as the influence of air moisture on the sample.

As reported previously, the *L*-serine monohydrate phase is not stable under MAS [S1]. We thus recorded time-dependent ^{13}C CP-MAS spectra using a freshly filled rotor with *L*-serine monohydrate. The results are shown in Figure S9 and indeed reveal a decrease in the *L*-serine monohydrate resonances and the appearance of new resonances assigned to *L*-serine.

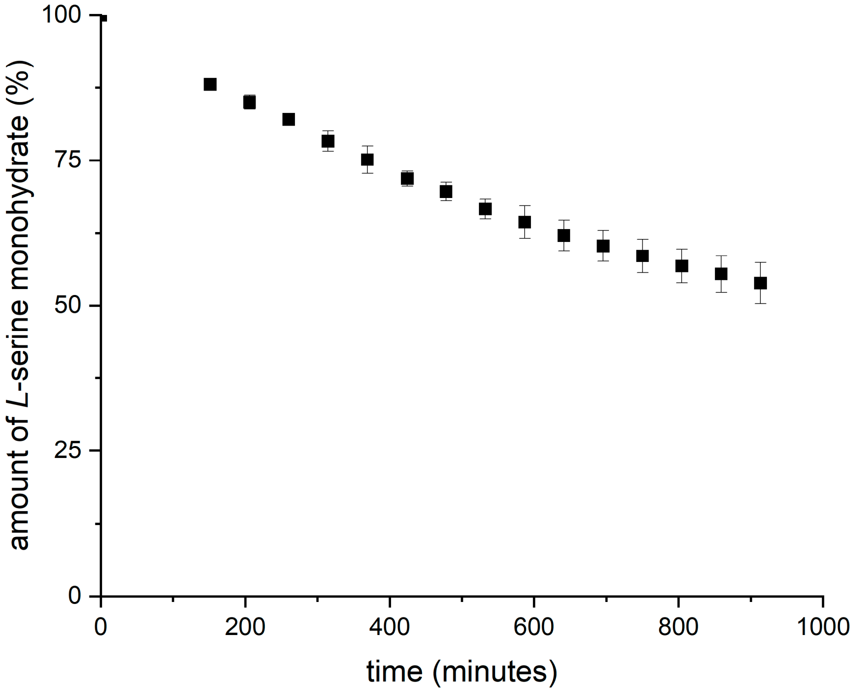

Figure S9. The correlation between the amount of *L*-serine monohydrate present in the sample over time. The error bars are calculated as the standard deviation of the amounts of racemic amino acid gained from the integration of the NMR signals.

The results show an exponential decay of the *L*-serine monohydrate phase. This effect has been observed before with the monohydrate phase completely decaying in approximately 500 minutes [S1]. In our setup, the decay is significantly slower which may be due to differences in the experimental parameters such as temperature or a difference in the preparation of the monohydrate phase.

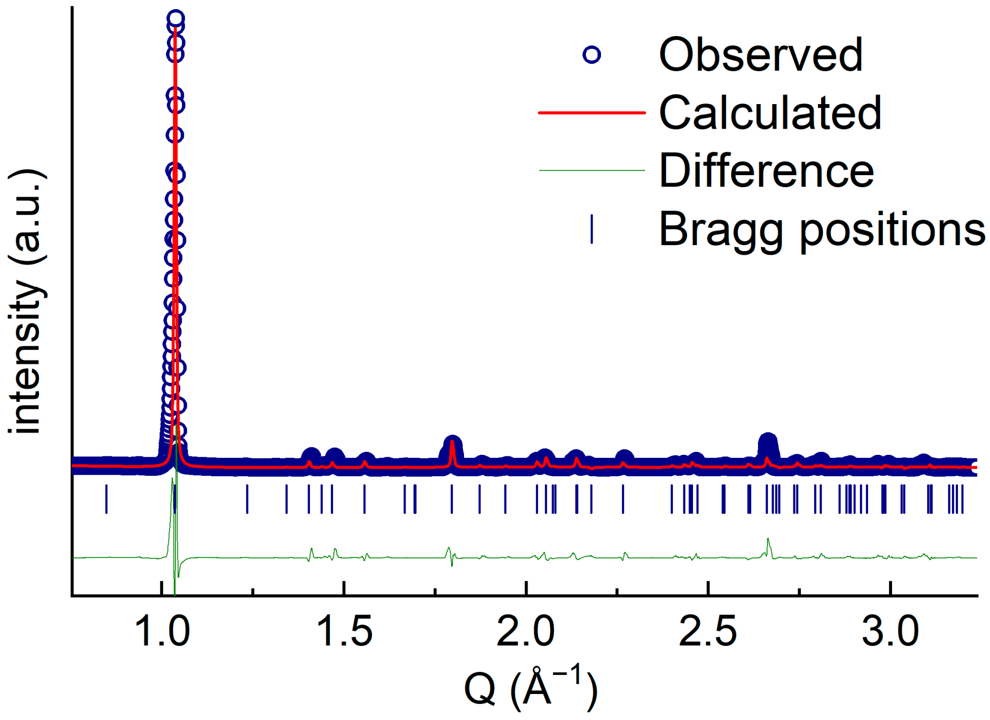

Figure S10: Rietveld refinement of PXRD measurement of *L*-serine monohydrate samples prepared in the resonant acoustic mixer. The deviations from the fit can be attributed to the strong preferred orientation of serine as well as the sample reverting to the anhydrous serine phase over time during the measurement.

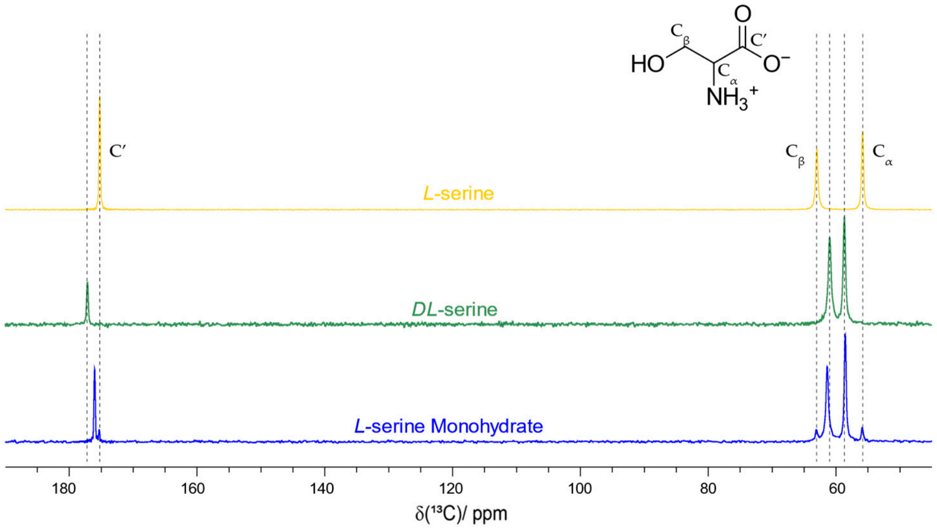

Figure S11. ^{1}H-^{13}C CP-MAS spectra of purchased *L*-serine (yellow) as well as purchased *DL*-serine (green) and *L*-serine monohydrate formed in the resonant acoustic mixer (blue). The less intense resonances in the *L*-serine monohydrate spectrum (blue) point to a residual amount of water-free *L*-serine in the sample. All spectra were recorded at 11.7 T, 285 K and 17.0 kHz MAS frequency.

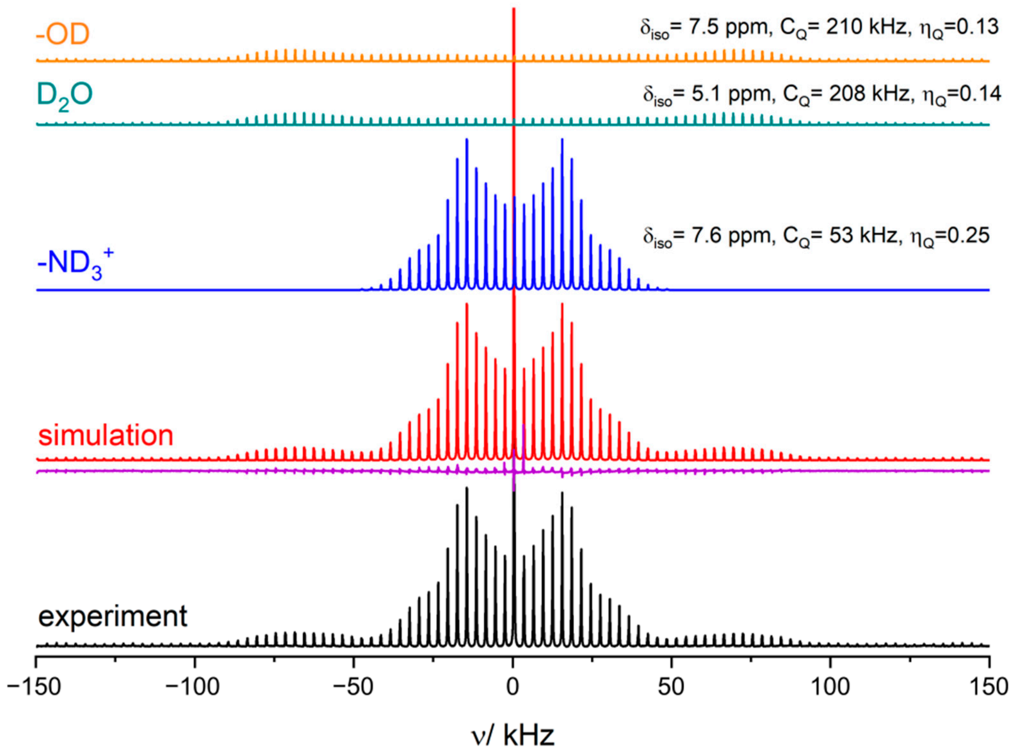

Figure S12. [2]D MAS spectrum (black) and simulations of *L*-serine monohydrate. The spectrum was recorded at 11.7 T, 285 K and 3.0 kHz MAS frequency. The simulations were performed using DMFit [S2]. Individual contributions to the line shape are plotted as colored lines, and the purple line represents the difference between the experimental and simulated spectrum.

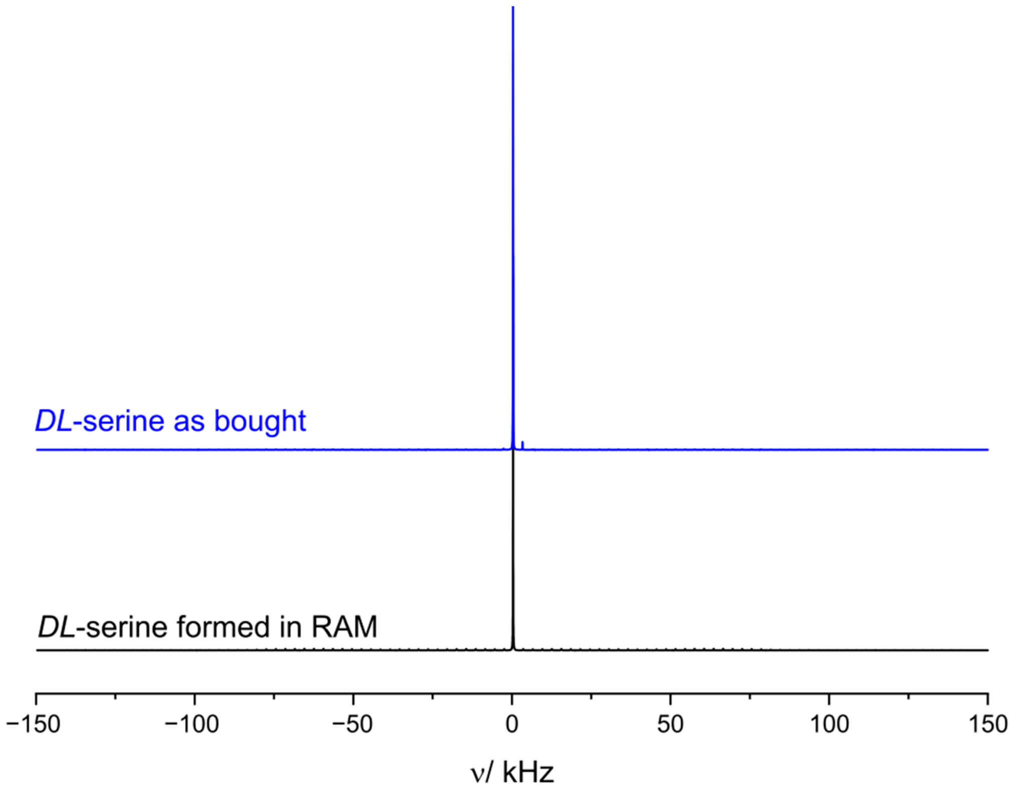

Figure S13. ^{2}D MAS spectrum of DL-serine created by combining D- and L-serine with D_2O in the resonant acoustic mixer (black) and DL-serine processed with D_2O in the resonant acoustic mixer (blue). The spectra were recorded at 11.7 T, 285 K and 3.0 kHz MAS frequency.

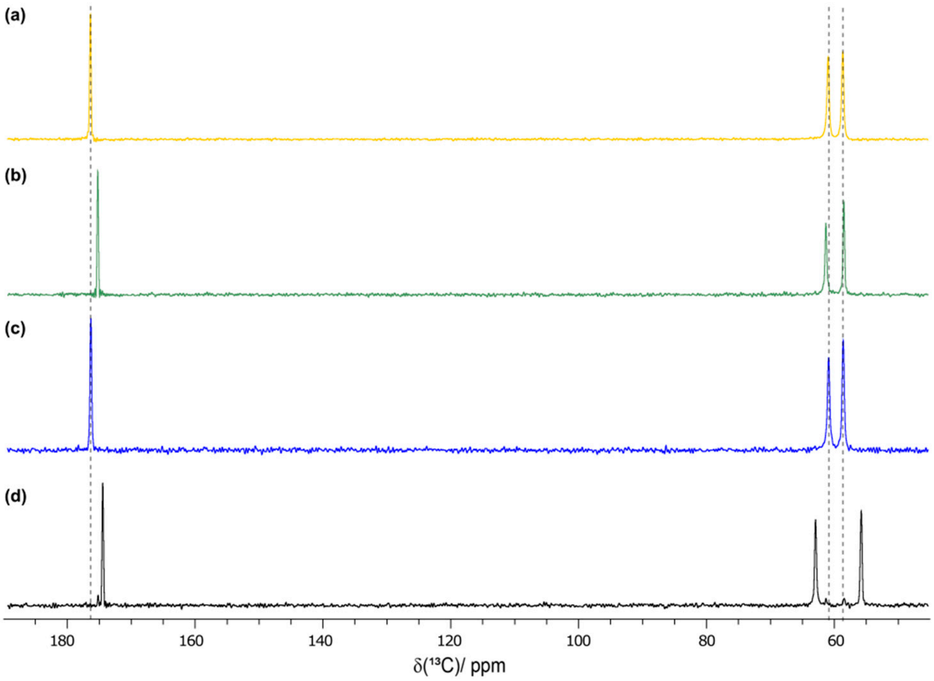

Figure S14. ^{1}H-^{13}C CP-MAS spectra of (a) *L*- and *D*-serine mixed with a spatula, (b) *L*-serine monohydrate on the left side of the contact surface, (c) material from the contact surface between *L*- and *D*-serine, and (d) *D*-serine monohydrate and anhydrous *D*-serine from the right side of the contact surface. The samples were stored in a desiccator at >95% humidity for approximately 3 days. All spectra were recorded at 11.7 T, 285 K and 17.0 kHz MAS frequency. Dashed lines highlight *DL*-serine resonances. The expected monohydrate phase of D-serine in sample (d) has mostly transformed back into its anhydrous phase due to the time delay between taking the entire sample out of the desiccator and the start of measurement.

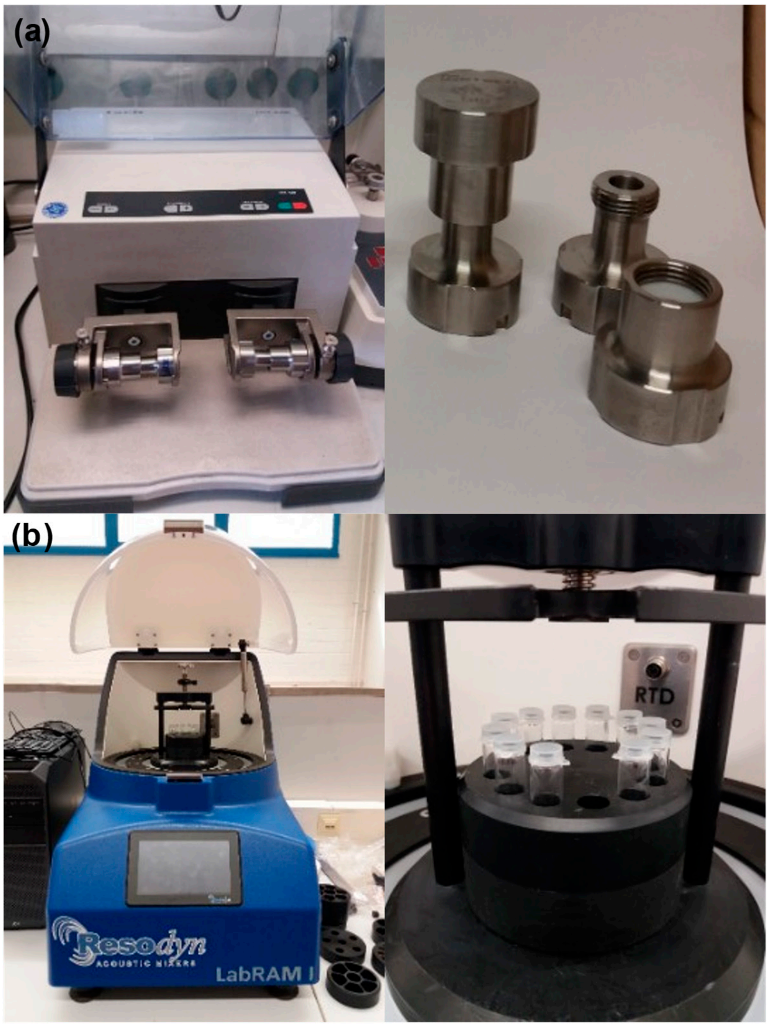

Figure S15. Images of (**a**) the MM400 shaker mill with its milling vessels to the right and (**b**) the LabRAM I with the custom-made vial carrier to its right.

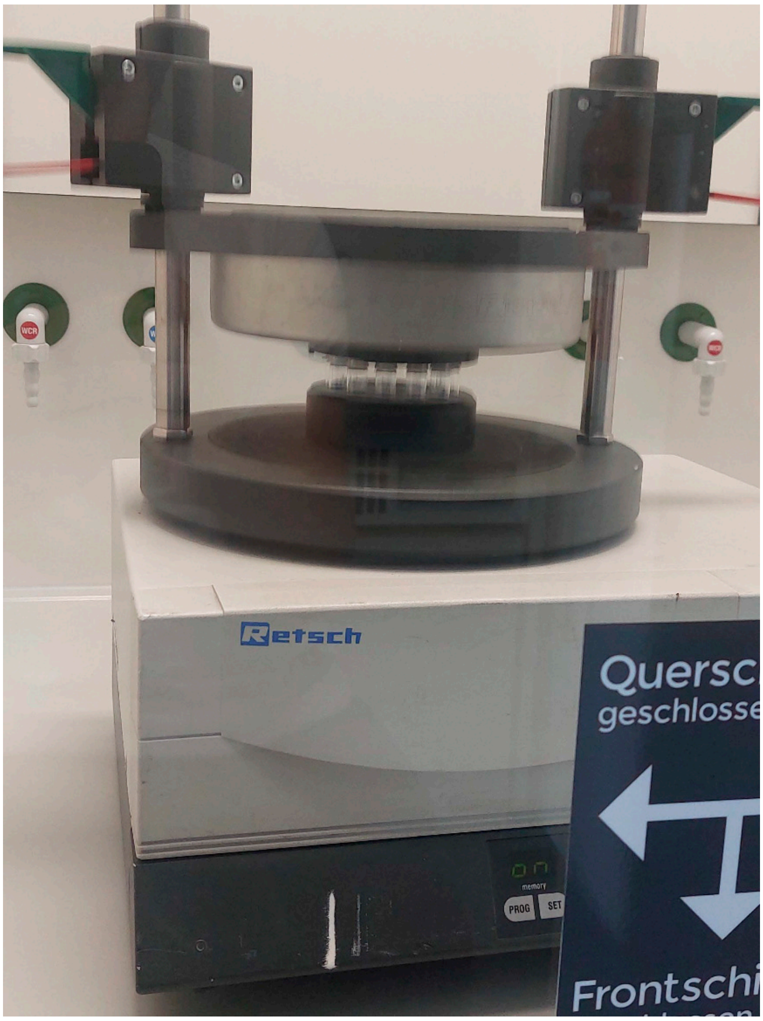

Figure S16. Images of the AS200 basic vibratory sieve shaker with the sample holder clamped in between the vibration plate and a sample pan.

Table S5. Experimental parameters of the conducted ^{1}H-^{13}C CP MAS experiments.

**Sample**	**Serine**	**Alanine**
**Experiment**	**^{1}H-^{13}C CP-MAS**	
υ_r/kHz	17.0	
B_0/T	11.7	
**CP polarization Transfer**	**H-C CP**	
$\upsilon_1(^{1}H)$/kHz	61.3	
$\upsilon_1(^{13}C)$/kHz	44.6	
Shape	Tangent shape	
^{13}C carrier/ppm	100	
CP contact time/µs	1500	400
Sweep width/ppm	795.1	
Acquisition time/ms	25.6	
^{1}H Spinal64 decoupling/kHz	90	
Number of scans	32	256
Interscan delay/s	90	5
Measurement time/min	49	22
Probe target temperature/K	285	

Work procedures

- Preparation of samples with different *ee*-values

  The samples were prepared by mixing *L*-serine with *DL*-serine and *L*-alanine with *DL*-alanine, respectively, to achieve four samples each with 20% *ee*, 40% *ee*, 60% *ee* and 80% *ee* contents. An amount of 10 mg of the enantiopure amino acids was combined with 40 mg of the respective racemic amino acids to prepare the 20% *ee* sample; 20 mg of enantiopure amino acids were combined with 30 mg of the respective racemic amino acids to prepare the 40% *ee* sample; 30 mg of enantiopure amino acids were combined with 20 mg of the respective racemic amino acids to prepare the 60% *ee* sample; and 40 mg of enantiopure amino acids were combined with 10 mg of the respective racemic amino acids to prepare the 80% *ee* sample.

- Calibration of parameters for serine/alanine measurements

  Calibrations were performed for both serine and alanine. To this end, measurements were performed on multiple samples of known ratios between racemic and *L*-amino acid phases. The ratios used were 20% ee, 40% ee, 60% ee, and 80% ee. These were measured while optimizing the measurement parameters, and the ratios calculated from the NMR spectra were compared to the weighed-in ratios to create a calibration curve. The results are shown in Figure 1b and 1d.

  The datapoints were created by calculating the ratio for every signal pair separately and afterwards taking the average across all three values. During the first attempts, this resulted in major deviations for alanine due to the high inconsistency of the signal pair around 50 ppm. It was determined that even with the help of "DMFit", separating the two signals was not accurate enough and this signal pair was not taken into account for the calculated values shown in Figure 1d. The values obtained from the measurements were plotted against the weighed in contents of the samples. A linear fit was applied to the dataset and the implied points of 0,0 and 100,100 were added for an easier visual assessment. Serine follows the linear fit very well, meaning the values calculated from NMR using the optimized measurement parameters could be used without any modification. Alanine, on the other hand, shows minor deviations mostly in samples with lower amounts of enantiopure phase. It was debated if a correction factor should be applied to the results calculated from NMR to account for these deviations, but due to the inconsistency in the deviation itself, it was determined that the results obtained from the optimized parameter set for alanine could also be used without further modification.

- Preparation of LA-RAM alanine samples with a variation on solvents

  Five samples were prepared using different solvents during the mixing process. A quantity of 25 mg of *L*-alanine was combined with 25 mg of *D*-alanine and 10 µL of solvent ($\eta = 0.2\,\mu L/mg$) in a 2 mL snap cap vial. The solvents used were acetonitrile, dimethyl sulfoxide, ethanol, and deionized water. The samples were than processed in the resonant acoustic mixer for 20 minutes at 100 *g*.

- Preparation of LA-RAM samples with a variation on η-value in the resonant acoustic mixer

  Six samples were prepared by changing the η-value used. A quantity of 25 mg of *L*-serine/*L*-alanine was combined with 25 mg of their respective counterpart *D*-

serine/*D*-alanine and a variable amount of deionized water in a 2 mL snap cap vial. The amounts used were 2.5 μL (η = 0.05 μL/mg), 5 μL (η = 0.1 μL/mg), 10 μL (η = 0.2 μL/mg), 15 μL (η = 0.3 μL/mg), 20 μL (η = 0.4 μL/mg) and 25 μL (η = 0.5 μL/mg). The samples were than processed in the resonant acoustic mixer for 20 minutes at 100 *g*.

- Preparation of LA-RAM samples with a variation on η-value in the AS200

  Six samples were prepared by changing the η-value used. A quantity of 25 mg of *L*-serine was combined with 25 mg of *D*-serine and a variable amount of deionized water in a 2 mL snap cap vial. The amounts used were 2.5 μL (η = 0.05 μL/mg), 5 μL (η = 0.1 μL/mg), 10 μL (η = 0.2 μL/mg), 15 μL (η = 0.3 μL/mg), 20 μL (η = 0.4 μL/mg) and 25 μL (η = 0.5 μL/mg). The samples were than processed in the AS200 for 20 minutes at 25 *g*.

- Preparation of the *L*-serine monohydrate sample

  An amount of 50 mg of *L*-serine was combined with 10 μL of deionized water (η = 0.3 μL/mg) in a 2 mL snap cap vial. The sample was than processed in the resonant acoustic mixer for 20 minutes at 100 *g*.

- Preparation of *L*-serine monohydrate and racemic serine samples using D_2O

  Two samples were prepared with deuterium oxide. For the first sample, 50 mg of *L*-serine was combined with 15 μL of deuterium oxide (η = 0.3 μL/mg) in a 2 mL snap cap vial. For the second sample, 25 mg of *L*-serine was combined with 25 mg of *D*-serine and 15 μL of deuterium oxide (η = 0.3 μL/mg) in a 2 mL snap cap vial. The samples were than processed in the resonant acoustic mixer for 20 minutes at 100 *g*.

- Analysis of serine and alanine NMR spectra

  To obtain the percentage values of *DL*-amino acids from the spectra, enantiopure and *DL*-amino acid resonances associated with the same chemical group in the molecule were integrated and the relative ratios were determined.

  For serine, the averages of the three obtained ratios are reported in this paper and the error bars were calculated as the standard deviation of the amounts of *DL*-serine gained from the integration of the three NMR resonances.

  For alanine, the averages of the C′ and $C_ß$ resonance ratios are reported in this paper and the error bars were calculated as the standard deviation of the amounts of *DL*-alanine gained from the integration of the two NMR resonances. Due to overlapping resonances in the ^{13}C MAS spectra of alanine, the software DMfit version:"x64/release#20230120" was used for line shape simulation and subsequent integration [S2].

- Simulation of D_2O spectra

  The software DMfit version:"x64/release#20230120" was used to simulate the D2O spectra. It was used to correct the baseline, create the line shape and calculate the quadrupolar coupling constants as well as the asymmetry parameters [S2].

S1. Kameda, T.; Teramoto, H. Phase transition of L-Ser monohydrate crystal studied by ^{13}C solid-state NMR. *Magn. Reson. Chem.* **2006**, *44*, 318-324, doi:10.1002/mrc.1745.
S2. Massiot, D.; Fayon, F.; Capron, M.; King, I.; Le Calvé, S.; Alonso, B.; Durand, J.O.; Bujoli, B.; Gan, Z.; Hoatson, G. Modelling one- and two-dimensional solid-state NMR spectra. *Magn. Reson. Chem.* **2001**, *40*, 70-76, doi:10.1002/mrc.984.
